# BODIPY-doped silica nanoparticles with reduced dye leakage and enhanced singlet oxygen generation

**DOI:** 10.1038/srep12602

**Published:** 2015-07-27

**Authors:** Zhuyuan Wang, Xuehua Hong, Shenfei Zong, Changquan Tang, Yiping Cui, Qingdong Zheng

**Affiliations:** 1 Advanced Photonics Center, Southeast University, Nanjing 210096, P. R. China; 2 State Key Laboratory of Structural Chemistry, Fujian Institute of Research on the Structure of Matter, Chinese Academy of Sciences, 155 Yangqiao West Road, Fuzhou, 350002, P. R. China

## Abstract

Photodynamic therapy (PDT) is a promising modality for cancer treatment. The essential element in PDT is the photosensitizer, which can be excited by light of a specific wavelength to generate cytotoxic oxygen species (ROS) capable of killing tumor cells. The effectiveness of PDT is limited in part by the low yield of ROS from existing photosensitizers and the unwanted side effects induced by the photosensitizers toward normal cells. Thus the design of nanoplatforms with enhanced PDT is highly desirable but remains challenging. Here, we developed a heavy atom (I) containing dipyrromethene boron difluoride (BODIPY) dye with a silylated functional group, which can be covalently incorporated into a silica matrix to form dye-doped nanoparticles. The incorporated heavy atoms can enhance the generation efficiency of ROS. Meanwhile, the covalently dye-encapsulated nanoparticles can significantly reduce dye leakage and subsequently reduce unwanted side effects. The nanoparticles were successfully taken up by various tumor cells and showed salient phototoxicity against these cells upon light irradiation, demonstrating promising applications in PDT. Moreover, the incorporated iodine atom can be replaced by a radiolabeled iodine atom (e.g., I-124, I-125). The resulting nanoparticles will be good contrast agents for positron emission tomography (PET) imaging with their PDT functionality retained.

Photodynamic therapy (PDT) has been proven to be an effective treatment for easily accessible tumors, such as oral and skin cancers^1^,^2^,^3^,^4^,^5^. As an essential element for cancer treatment, photosensitizers (drugs) can react with oxygen and create a burst of reactive oxygen species (ROS) that kill tumor cells. Therefore, in the past two decades, the development of PDT has been associated with the invention of novel and effective photosensitizers^3^. The effectiveness of PDT has been limited in part by the low yield of reactive oxygen species from the existing photosensitizers and the unwanted side effects induced by the photosensitizers targeted at normal cells. The efficiency of singlet oxygen generation is proportional to the population of the excited triplet photosensitizers, which are produced by intersystem crossing (ISC) from the excited singlet photosensitizers. Therefore, the singlet oxygen generation efficiency of a photosensitizer (PS) can be enhanced by increasing the efficiency of ISC through strong spin-orbital coupling in the presence of heavy atoms (such as I, Br)^6^,^7^. So far, most PDT studies are based on traditional photosensitizers, such as porphyrins and phthalocyanines^8^,^9^,^10^,^11^. While studies concerning the heavy atom effect on the singlet oxygen generation as well as the resulting PDT efficiency are relatively rare^12^,^13^,^14^. Since the first successful synthesis of silica nanoparticles (NPs) by Mobil scientists, significant advances have been made in modifying and controlling the properties of the mesoporous silica materials^15^,^16^. Among them, the manipulation on optical properties of silica NPs is of vital importance because the pure silica NPs themselves barely absorb or emit photons in the visible and IR range in which most light sources, sensors and detectors are active^17^,^18^. Furthermore, various functional groups (hydroxyl/amino/thiol/carboxyl groups) can be incorporated into the silica surface of the nanoparticles for additional functionalities such as MR/radio imaging and a therapeutic application^19^,^20^. Therefore, silica matrices are highly attractive as the structural basis for a wide variety of nanotechnological applications, such as adsorption, sensing, and separation^17^,^21^,^22^. With the mesoporous feature of silica NPs, organic chromophores can be easily doped into them, which will impart the silica NPs with the optical properties of the chromophores. However, physical encapsulation of the organic chromophores cannot exclude the partial release of the chromophores over time upon the environmental changes. As we know, in the PDT application, selective targeting of PS on cancer cells is required in order to reduce the risk of unwanted side-effects. Therefore, photosensitizers covalently linkable to the silica matrix are needed and the resulting nanoparticles will have the advantage of avoiding the PS leakage.

Dipyrromethene boron difluoride (BODIPY) dyes are excellent fluorescent probes and their chemical structures can be modified to incorporate a number of functionalities^23^,^24^,^25^. In this context, we design a novel BODIPY dye with a silylated group and two heavy atoms (I). The silylated group is used to covalently encapsulate the dye into silica nanoparticles, and the iodine atoms are used to enhance the singlet oxygen generation of the resulting dye-doped silica NPs. The iodine atoms on the backbone of the silylated BODIPY dye can be used to study the heavy atom effect on the singlet oxygen generation as well as the resultant PDT application. Meanwhile, a silylated BODIPY dye without heavy atoms is used as the control. With the silylated group, it is expected that the dye leakage of silica NPs will be reduced, which is beneficial for achieving a decreased side effect in PDT.

## Results and Discussion

### Synthesis

**Figure 1:**
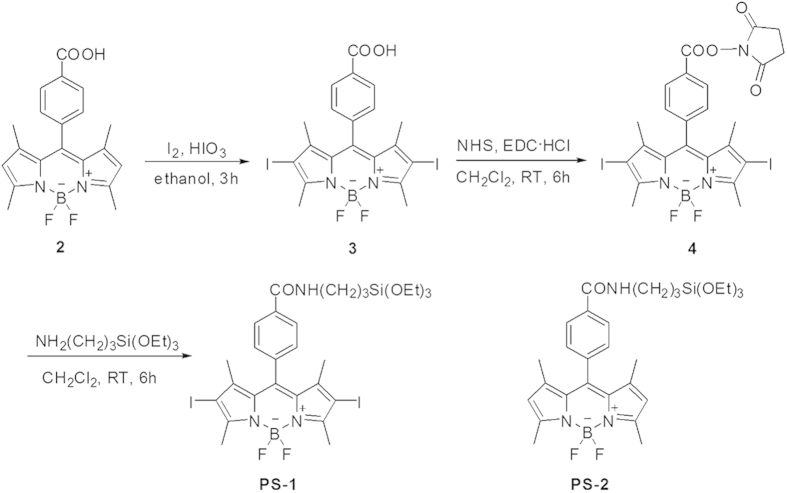
The synthetic route to **PS-1** and the chemical structure of **PS-2**.

The synthetic route to the target silylated BODIPY dye is depicted in Fig. 1. Compound **3** was synthesized from compound **2** through an iodination reaction in 88% yield. Then, the activation of the carboxyl group in compound **3** by reacting with N-hydroxysuccinimide (NHS) afforded compound **4** in 72%. Compound **4** was then reacted with (3-aminopropyl)triethoxysilane (APTES) to get the final compound **PS-1** in 61% yield. The synthesis of **PS-2** has been described by us previously^24^.

**Figure 2:**
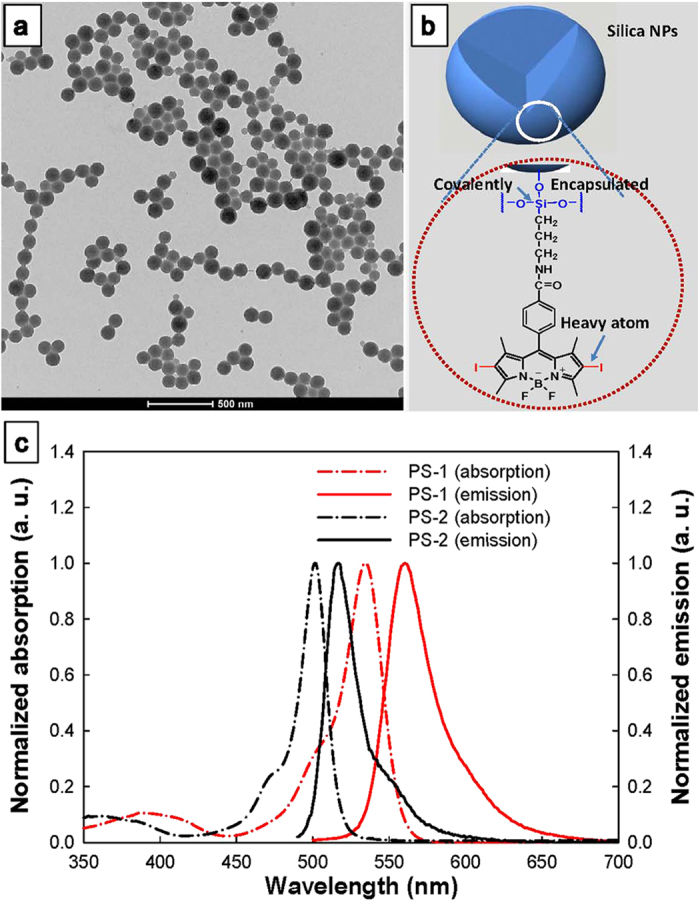
(**a**) TEM picture of the **PS-1**-doped silica NPs showing monodispersed particles with an average diameter of 85 nm; (**b**) The structure of **PS-1**-doped silica NPs; (**c**) Normalized linear absorption and emission spectra of compounds **PS-1** and **PS-2** in DMSO.

The silica NPs were synthesized by the alkaline hydrolysis and polycondensation of the organotrialkoxysilane precursors within the nonpolar core of the sodium dioctylsulfosuccinate (AOT)/water microemulsion. A TEM image of the **PS-1**-loaded nanoparticles is shown in Fig. 2. The particles are of spherical shape and have a uniform size distribution. The average diameter of the silica NPs is 85 nm.

### Linear absorption and emission

The linear absorption and emission spectra of 2,6-diiode substituted BODIPY (**PS-1**) were measured in DMSO at room temperature and the results are shown in Fig. 2c. For comparison, the absorption and emission spectra of the control dye (**PS-2**) were also measured and depicted in Fig. 2. **PS-1** has a linear absorption maximum at 534 nm and an emission peak at 560 nm. Compared to the control dye (**PS-2**) under the same condition, the absorption and emission peaks of **PS-1** red-shift by 32 nm and 43 nm, respectively. This means that the introduction of two iodine atoms to the BODIPY core leads to red shifts in both the absorption and fluorescence maxima, which can be attributed to the extended π-conjugation with two extra iodine atoms attached^26^. With the iodine atoms incorporated, the fluorescence quantum yield of **PS-1** is 1.3%. While for **PS-2** without iodine atoms, the fluorescence quantum yield is 75%. Thus, around 58-fold quench of the fluorescence was found for the iodine containing chromophore (**PS-1**), which is caused by the heavy atom effect^27^. The decreased fluorescence quantum yield indicates an increase of the intersystem crossing rate of **PS-1**, and thus an increased ^1^O_2_ generation.

**Figure 3:**
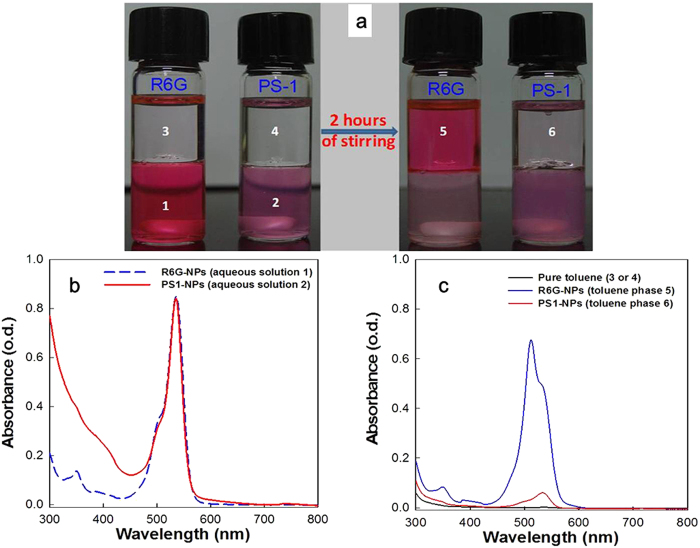
(**a**) Pictures of the physically dye-encapsulated (R6G) nanoparticles and covalently dye-encapsulated (**PS-1**) nanoparticles before and after stirring for 2 hours; (**b**) Absorption spectra for aqueous solutions of the physically dye-encapsulated (R6G) nanoparticles and covalently dye-encapsulated (**PS-1**) nanoparticles; (**c**) Absorption spectra of pure toluene, and toluene extractions from the physically dye-encapsulated (R6G) nanoparticles and covalently dye-encapsulated (**PS-1**) nanoparticles.

To find out whether the silylated BODIPY dye was covalently incorporated into nanoparticles rather than merely physically encapsulated, we mixed toluene with the aqueous nanoparticles solution (volume ratio = 1:1) to detect the dispersion of the dyes. Silica NPs with physically encapsulated Rhodamine 6G (R6G) were used as the control. Before stirring, the organic toluene phase was in the upper part of the vial, and it was colorless (Fig. 3a). After stirring for two hours, obvious dye leakage was observed for R6G-encapsulated nanoparticles and most of the R6G dyes were transferred into the organic phase (Toluene). From Fig. 3a, one can clearly observe the change in color due to the migration of R6G molecules from the aqueous phase to the organic phase. By contrast, dye leakage did not happen in the covalently **PS-1**-encapsulated nanoparticles (Fig. 3a). No visible color change in the toluene phase was observed. In order to quantitatively investigate the phenomenon, the absorption properties of these solutions before and after stirring were studied and the results are shown in Fig. 3b,c. As shown in the figure, before stirring, the peak absorption density of both particle solutions was tuned to an optical density of 0.82. After stirring, the peak absorption density of toluene phase increased to 0.68 for the R6G-encapsulated nanoparticles. On the contrary, the peak absorption density of toluene phase only increased to 0.10 for the **PS-1**-encapsulated nanoparticles, which is much lower than the physically encapsulated nanoparticles. The main reason for this phenomenon is that the dyes physically encapsulated in the nanoparticles can easily leak out from the porous silica matrix, move and dissolve into the toluene phase. However, for the silylated BODIPY dyes, the dye molecules are covalently linked to the silica matrix and little dye leakage was observed. As a result, these covalently dye-encapsulated nanoparticles exhibit advantages over physically dye-encapsulated nanoparticles in avoiding dye leakage, which would be of vital importance during *in vivo* circulation of photosensitizers.

### Singlet oxygen generation

**Figure 4:**
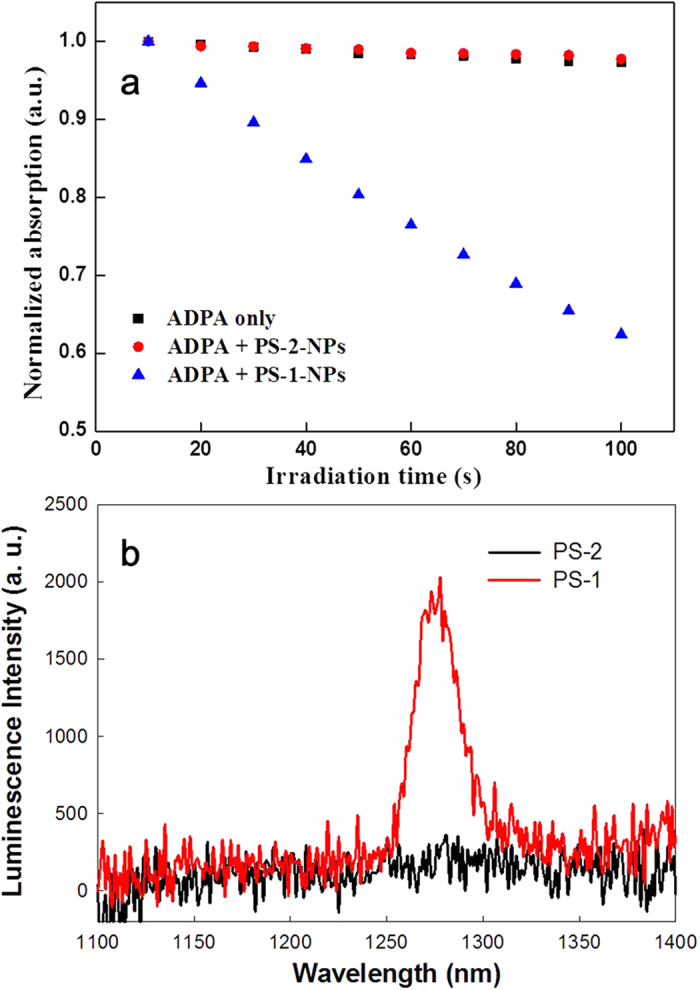
(**a**) Change of normalized optical intensity of ADPA’s absorption peak at 378 nm over time when the mixture containing ADPA and nanoparticles was irradiated by light. Neat ADPA water solution was used as a control; (**b**) Determination of the singlet oxygen generation efficiency for **PS-1** and **PS-2**.

In order to test the singlet oxygen (^1^O_2_) generation ability of the photosensitizer, anthracene-9, 10-dipropionic acid (ADPA) was generally selected as a molecular probe to assess the singlet oxygen production. After reaction with ^1^O_2_, ADPA is converted into an endoperoxide form, which will cause a decrease of the optical densities^28^. Here, we first added ADPA to the suspension of **PS-1**-NPs and **PS-2**-NPs, and subsequently recorded the time-dependent reduction of optical densities of ADPA at 378 nm. The result was shown in Fig. 4a. When the ADPA solution was mixed with **PS-1**-NPs, the optical densities of ADPA decreased over the light irradiation, indicating the generation of singlet oxygen. On the contrary, when mixed with the **PS-2**-NPs, the optical densities of ADPA nearly did not change, similar to the case of neat ADPA water solution where no singlet oxygen was generated (Fig. 4a). The results on singlet oxygen production clearly showed the enhanced generation of singlet oxygen by **PS-1**-conjugated nanoparticles in comparison with **PS-2**-conjugated nanoparticles. The enhanced release of singlet oxygen was caused by the introduction of iodine atoms, which is in agreement with the decreased fluorescence quantum yield of **PS-1**.

To further investigate the heavy atom effect in the BODIPY chromophores, ^1^O_2_ generation efficiencies of **PS-1** and **PS-2** in air-saturated toluene solution were evaluated by monitoring the characteristic emission peak of singlet oxygen at ~1270 nm at room temperature. As shown in Fig. 4b, under an excitation at 430 nm, **PS-1** displayed the characteristic ^1^O_2_ emission band at ~1270 nm, which confirmed that the ^1^O_2_ was generated from the interaction between the photosensitizers and molecular oxygen. However, under the same condition, **PS-2** did not exhibit the emission peak at ~1270 nm, reconfirming the important role of the heavy atom in affecting the ^1^O_2_ generation efficiency of a given chromophore. Therefore, **PS-1**-congugated nanoparticles were used for studying the *in vitro* photosensitizing activity in different cancel cells.

### Cytotoxicity against cancer cells

To evaluate **PS-1** as an effective photosensitizer for PDT of tumors, we performed *in vitro* photosensitizing experiments using three different cancer cells. The cancer cells are human cervical cancer cells (HeLa) and human breast cancer cells (MCF-7 and SKBR-3). To better prove that the fabricated NPs can be taken up by live cancer cells, we performed fluorescence imaging and Z-sliced imaging of HeLa cells incubated with **PS-1**-NPs using the fluorescence signals of **PS-1**. The results are shown in the Supplementary Fig. S1 and Fig. S2, which well confirmed that **PS-1**-NPs have been successfully taken up by live cells.

**Figure 5:**
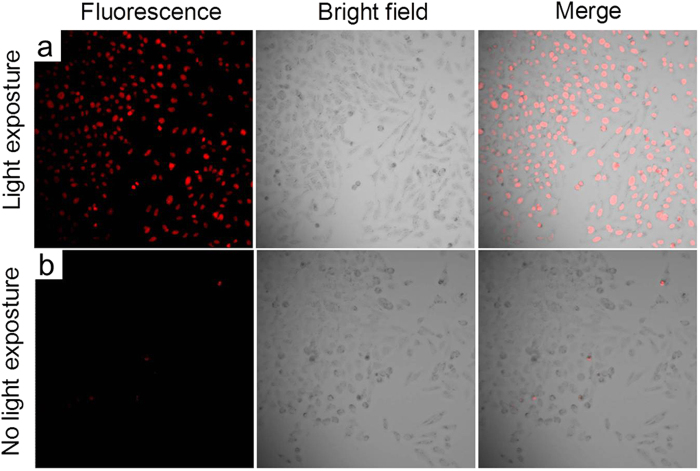
HeLa cell imaging using **PS-1-**NPs with (**a**) and without (**b**) light irradiation. The left panel is fluorescence channel of PI, the middle panel is bright field images of the HeLa cells, and the right panel is the merged images.

**Figure 6:**
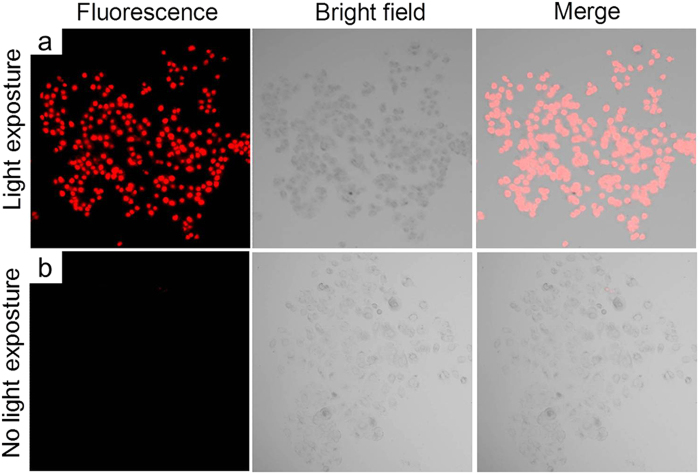
MCF-7 cell imaging using **PS-1**-NPs with (**a**) and without (**b**) light irradiation. The left panel is fluorescence channel of PI, the middle panel is bright field images of the MCF-7 cells, and the right panel is the merged images.

**Figure 7:**
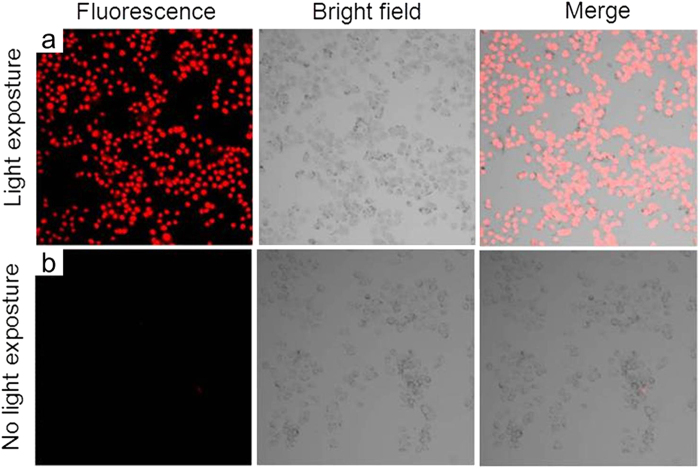
SKBR-3 cell imaging using **PS-1**-NPs with (**a**) and without (**b**) light irradiation. The left panel is fluorescence channel of PI, the middle panel is bright field images of the SKBR-3 cells, and the right panel is the merged images.

Next, in the PDT experiment, the cancer cells were first incubated with **PS-1** NPs to allow sufficient cellular uptake of the nanoparticles. Then Propidium Iodide (PI) was added into the cancer cell culture dishes to facilitate the investigation of cell viability. PI is a fluorescent vitality dye that does not stain the cell unless the cell membrane is damaged. Subsequently, the culture dishes containing these cancer cells were exposed to an argon laser beam (514 nm, 12.7 W/cm^2^, 3 minutes) with a light dose of 2291.8 J/cm^2^. After the laser illumination, the culture media were replaced by PBS buffer and the culture dishes were mounted onto an inverted confocal microscope. Fluorescence images of the cancer cells were taken to monitor the cell viability. Images of cancer cells without light irradiation and with light irradiation are shown in Figs 5, 6, 7, respectively. As shown in the figures, all three different cancer cells exposed to the 514 nm laser were stained by PI, which suggests the light induced cell death. We also used cancer cells without **PS-1** incubation as an additional control, while no cell death was found with the same light dose (data not shown).

**Figure 8:**
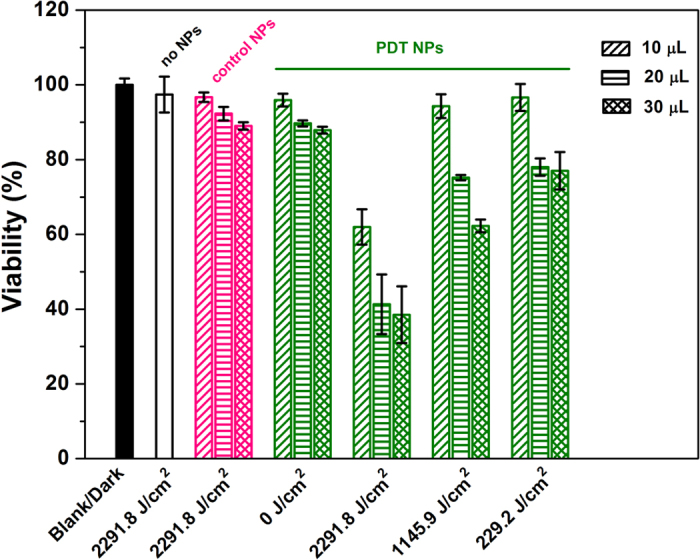
Viability of SKBR-3 cells incubated with different concentrations of nanoparticles and exposed to different light doses.

To quantitatively evaluate and compare the *in vitro* cytotoxicity of the **PS-1**-NPs, and **PS-2**-NPs (control) with or without irradiation by the 514 nm laser, the viabilities of cells were evaluated by MTT assays. SKBR-3 cells were incubated with **PS-1**-NPs and **PS-2**-NPs, or without nanoparticles. As shown in Fig. 8, in the absence of the laser, the increase in the concentration of NPs only lead to a small decrease in cell viability. In contrast, **PS-1-**NPs under laser irradiation showed obvious cytotoxicity to SKBR-3 cells due to the enhanced singlet oxygen generation. From Fig. 8, it is found that the viability of SKBR-3 cells decreased with an increasing light dose. When SKBR-3 cells were incubated with **PS-1-**NPs (30 μL), 77.8 ± 6.2% cell viability was observed under a light dose of 229.2 J/cm^2^. When SKBR-3 cells were incubated with the same concentration of **PS-1-**NPs with larger light doses of 1145.9 J/cm^2^ and 2291.8 J/cm^2^, cell viabilities of 62.2 ± 2.2% and 38 ± 7.2% were observed respectively. However, under the same light dose of 2291.8 J/cm^2^, negligible cell death was detected for SKBR-3 cells incubated without any NPs or with the control NPs. Besides, we found that without the singlet oxygen generation enhancing ability, the control **PS-2**-NPs require a much larger light dose to kill the cells (see Supplementary Fig. S3). These results indicate that efficient PDT of cancer cells can be fulfilled using the **PS-1-**NPs.

## Conclusion

We have successfully designed and prepared a heavy atom (I) containing chromophore as well as the resulting dye-doped silica nanoparticles. An iodine free chromophore was also prepared for comparison. The results clearly demonstrated that the incorporation of heavy atoms leads to enhanced generation of singlet oxygen, which has been verified by a chemical probe method as well as a direct measurement of singlet oxygen luminescence. These nanoparticles can be successfully taken up by various tumor cells and showed phototoxicity toward the cells treated with light irradiation, demonstrating a great potential for PDT. Worth mentioning is that the iodine atom of the BODIPY can also be replaced by a radiolabeled iodine atom (e.g., I-124, I-125, etc.), and the resulting nanoparticles with their PDT functionality retained could be employed as good contrast agents for positron emission tomography imaging. Furthermore, the silica surface of the dye-doped nanoparticles is available for surface modification and bioconjugation, indicating that the presented silica NPs have a great potential in developing multifunctional therapeutic nanoplatforms.

## Methods

### Materials and instruments

All chemicals were purchased from Aladin (China) and Aldrich and were used without further purification. All solvents were of analytical grade and used as received. ^1^H NMR (400 MHz) was recorded on a Bruker AVANCE 400 spectrometer. The linear absorption and emission spectra of free dye and dye-NPs were recorded by using a spectrophotometer (Lambda 35 UV/vis) and a Cary Eclipse spectrophotometer, respectively. Morphology of the nanoparticles was analyzed by using transmission electron micrograph (TEM).

### Synthesis of compound 3

Compound **2** (0.5 g, 1.36 mmol), iodine (0.863 g, 3.40 mmol) and iodic acid (0.473 g, 2.72 mmol) were dissolved in ethanol (120 mL). A few drops of water was added to the solution. This reaction mixture was stirred at 60 °C for about 3 h. After completion of the reaction, the ethanol was distilled off under reduced pressure. Then the residue was extracted with ethyl acetate. The organic phase was dried over MgSO_4_ and the solvent was distilled off to afford compound **3** (0.741 g, 88%). ^1^H NMR (400 MHz, DMSO, 25 °C, TMS): *δ* = 8.13 (d, *J* = 8.0 Hz, 2H), 7.57 (d, *J* = 8.0 Hz, 2H), 2.60 (s, 6H), 1.34 ppm (s, 6H). MS: m/z: calcd for C_20_H_17_BF_2_N_2_O_2_I_2_: 619.9; found: 619.9.

### Synthesis of compound 4

The synthesis of compound **4** was similar to the method reported in previous literatures^24^. Briefly, N-hydroxysuccinimide (NHS) (0.223 g, 1.94 mmol) and 1-ethyl-3-(3-dimethyllaminopropyl)carbodiimide hydrochloride (EDC·HCl) (0.381 g, 1.94 mmol) was added to a solution of compound **3** (0.400 g, 0.645 mmol) in CH_2_Cl_2_. The resulting mixture was stirred for 6 h at room temperature. After reaction, the solution containing the crude product was extracted with CH_2_Cl_2_. The solvent was evaporated and the crude product was purified by column chromatography (silica gel, diethyl ether: hexane = 2:5) to produce compound **4** (0.333 g, 72%). ^1^H NMR (400 MHz, CDCl_3_, 25 °C, TMS): δ = 8.33 (d, *J* = 8.0 Hz, 2H), 7.51 (d, *J* = 8.0 Hz, 2H), 2.99 (brs, 4H), 2.68 (s, 6H), 1.42 ppm (s, 6H). MS: m/z: calcd for C_24_H_20_BF_2_N_3_O_4_I_2_: 717.0; found: 717.0

### Synthesis of PS-1

Under a nitrogen atmosphere, compound **4** (0.200 g, 0.279 mmol) and (3-aminopropy)triethoxysilane (APTES) (0.182 g, 0.837 mmol) in CH_2_Cl_2_ were stirred for 6 h at room temperature. The solution was concentrated under reduced pressure and purified by column chromatography (silica gel, diethyl ether: hexane = 2:5) to obtain the final **PS-1** (0.14 g, 61%). ^1^H NMR (400 MHz, CDCl_3_, 25 °C, TMS): *δ* = 7.97 (d, *J* = 8.0 Hz, 2H), 7.37 (d, *J* = 8.0 Hz, 2H), 3.85 (q, *J* = 6.8 Hz, 6H), 3.53 (q, *J* = 6.8 Hz, 2H), 2.65 (s, 6H), 1.86-1.79 (m, 2H), 1.38 (s, 6H), 1.24 (t, *J* = 7.2 Hz, 9H), 0.764 ppm (t, *J* = 8.0 Hz, 2H). HRMS: m/z: calcd for C_29_H_38_BF_2_N_3_O_4_I_2_Si: 823.1; found: 823.1

### Synthesis of PS-1-covalently incorporated silica nanoparticles

The nanoparticles were prepared by the hydrolysis and polycondensation of the organotrialkoxysilane precursors within the nonpolar core of oil in water microemulsion. 300 μL of co-surfactant 1-butanol was dissolved into 10 mL of 2% aqueous Tween-80 solution, forming an oil-in-water microemulsion. To this microemulsion, 100 μL of DMSO solution containing **PS-1** (10 mM) was added, followed by the addition of 100 μL of vinyl triethoxysilane (VTES), and the resulting mixture was stirred for an hour. Then the polymerization reaction was initialized by the addition of 10 μL of APTES. The solution was further stirred at room temperature overnight. Next, the surfactant, co-surfactant and other unreacted molecules were removed by using a dialysis cellulose membrane. Following dialysis, the nanoparticles were sterile filtered and stored at 4 °C for future use. The control sample was synthesized in the same way mentioned above except that **PS-1** was replaced by the control dye.

## Detection of singlet oxygen

The generation of singlet oxygen was monitored by using the disodium salt of 9, 10-anthracenedipropionic acid (ADPA) as the singlet oxygen capture agent. In a typical experiment, 1.5 mL of ADPA (1 × 10^−4^ M) was mixed with 1.5 mL of **PS-1**-NPs (1 × 10^−5^ M) suspension. In the control experiment, ADPA was mixed with the suspension of the control dye-NPs. Another control solution was prepared which contains ADPA alone. These three solutions were irradiated with simulated solar light (λ > 400 nm) and their optical densities at 378 nm were recorded every 10 seconds in a spectrophotometer (Lambda 35 UV/vis). ^1^O_2_ generation efficiencies of compounds **PS-1** and **PS-2** in air-saturated toluene solution were evaluated by monitoring the characteristic emission peak of singlet oxygen at ~1270 nm at room temperature. The concentrations of the solutions were adjusted to give an optical density (OD) of 0.5 at 430 nm.

## Cell experiments

Human cervical cancer cells (HeLa), human breast cancer cells (MCF-7 and SKBR-3) were purchased from China Type Culture Collection. HeLa and SKBR3 cells were cultured in Dulbecco’s Modified Eagle’s Medium (DMEM) and MCF-7 cells were cultured in Roswell Park Memorial Institute (RPMI) 1640 medium. Cells were kept under standard cell culture condition (5% CO_2_, 37 °C). Media were supplemented with 10% fetal bovine serum (GIBCO) and 1% penicillin–streptomycin (Nanjing KeyGen Biotech. Co., Ltd.). Cells were seeded into culture dish (Corning) and incubated for 24 h. Then the nanoparticles were added to the cell culture dish (volume ratio nanoparticles solution: culture media = 1:5) and incubated for 5 h. Then the cells were exposed to light irradiation and fluorescence measurements.

The viability of cells was examined by the MTT (3-(4,5-dimethylthiazol-2-yl)-2, 5-diphenyltetrazolium bromide) assay. SKBR3 cells (10^4^/mL) were seeded onto a 96-well plate (100 μL/hole) and incubated for 24 h at 37 °C under a 5% CO_2_ atmosphere. Then the solutions of the nanoparticles were added respectively and incubated for 5 h. After that, the cells were exposed to different doses of light irradiation and further incubated for 24 h. Then 50 μL of MTT solution (MTT buffer to dilution buffer 1:4) was added into each well and the plate was incubated for another 4 h. The reaction was terminated by adding 150 μL of DMSO after removing the supernatant medium. When the purple formazan crystals were dissolved by DMSO, the absorbance of the wells at 490 nm were measured with a microplate reader (Bio-Rad model 680). Cells incubated in the absence of nanoparticles were used as a control.

## Additional Information

**How to cite this article**: Wang, Z. *et al.* BODIPY-doped silica nanoparticles with reduced dye leakage and enhanced singlet oxygen generation. *Sci. Rep.* **5**, 12602; doi: 10.1038/srep12602 (2015).

## Supplementary Material

Supplementary Information
