# Supplementary material for: BODIPY-doped silica nanoparticles with reduced dye leakage and enhanced singlet oxygen generation
Source: Sci Rep. 2015 Jul 27;5:12602. doi: 10.1038/srep12602 (PMC4515827; doi:10.1038/srep12602)
Supplement: Supplementary Information [file srep12602-s1.pdf]

Supporting Information

BODIPY-doped silica nanoparticles with reduced dye leakage and enhanced singlet oxygen generation

Zhuyuan Wang,[a] Xuehua Hong,[b] Shenfei Zong,[a] Changquan Tang,[b] Yiping Cui*[a] and Qingdong Zheng[b]

[a]*Advanced Photonics Center, Southeast University, Nanjing 210096, P. R. China*
E-mail: Yiping Cui (cyp@seu.edu.cn)

[b]*State Key Laboratory of Structural Chemistry, Fujian Institute of Research on the Structure of Matter, Chinese Academy of Sciences, 155 Yangqiao West Road, Fuzhou, 350002, P. R. China*

Fluorescence imaging of HeLa cells using fluorescence signals of PS-1-NPs

To better prove that the fabricated nanoparticles (NPs) have been taken up by living cells. We have conducted fluorescent cell imaging using confocal laser scanning microscope. HeLa cells were first incubated with **PS-1**-NPs for 5 h. Then the culture medium containing excess NPs was discarded and the cells were subjected to fluorescence imaging using 543 nm laser excitation. Fluorescence signals (555-585 nm) originated from **PS-1** in the **PS-1**-NPs were collected for imaging. The results are shown in Figure S1. As can be seen, strong fluorescence signals were observed on these HeLa cells, which indicated that **PS-1**-NPs have been successfully taken up by live HeLa cells.

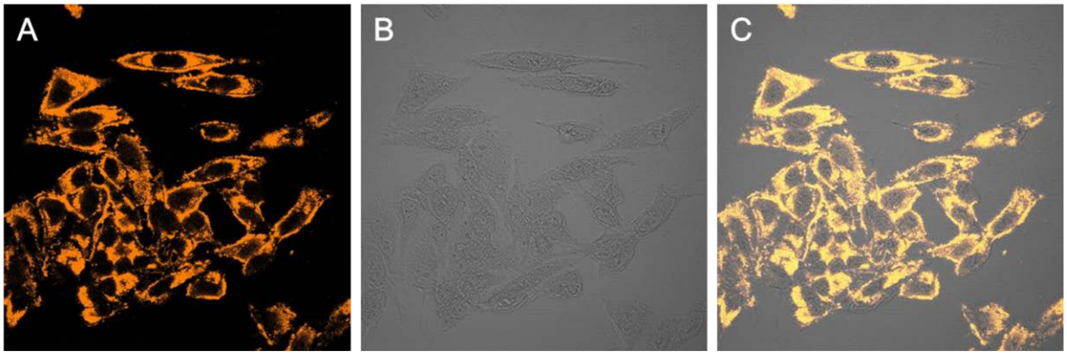

Figure S1. (A) Fluorescence image of HeLa cells incubated with **PS-1**-NPs. (B) Differential interference contrast (DIC) image of the same HeLa cells. (C) Merged image of (A) and (B).

Next, to better prove that the **PS-1**-NPs were incorporated into the cells, Z-sliced fluorescence images of these HeLa cells were acquired and the results are shown in Figure S2. Obviously, fluorescence signals were detected in the whole cellular plasma region, except for the nucleus region. This is reasonable since the relatively big NPs cannot pass through the nuclear membranes. The Z-slice results more vividly confirmed that **PS-1**-NPs were indeed taken up by live cells.

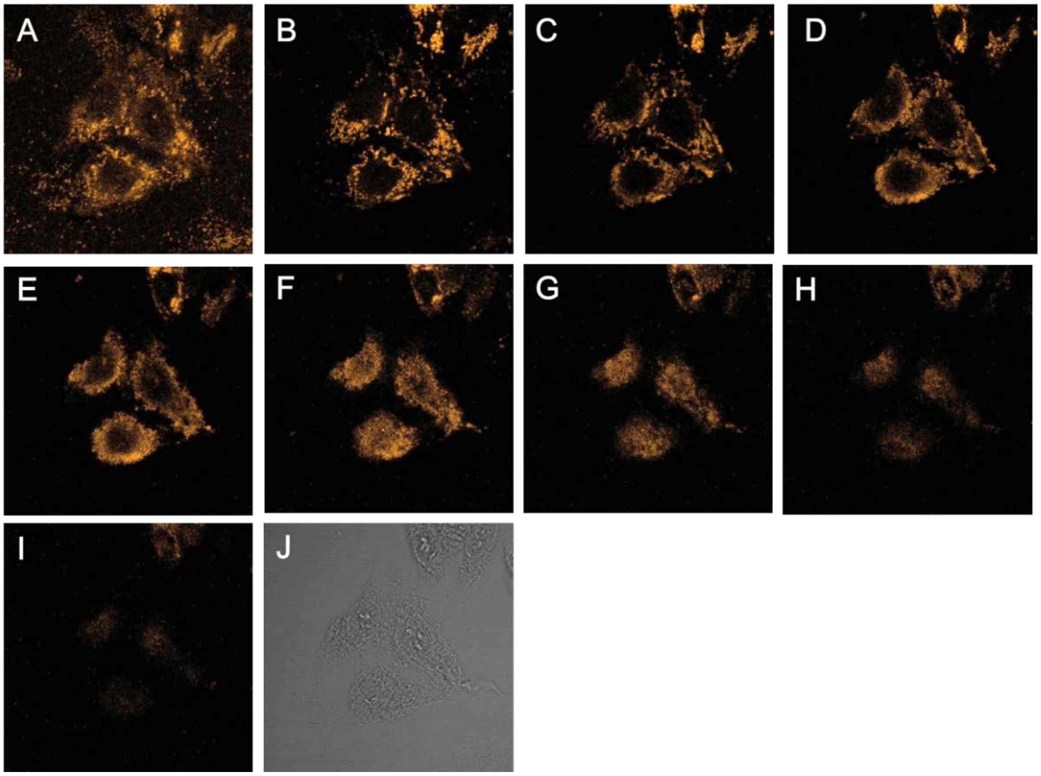

Figure S2. Z-sliced fluorescence images of HeLa cells incubated with **PS-1**-NPs with an interval of 1 µm. The Z position of the interface between HeLa cells and the cell culture dish was designated zero. (A-I) Z=0, 1, 2, 3, 4, 5, 6, 7, 8 µm. (J) DIC image of the same HeLa cells.

PDT of HeLa cells using PS-2-NPs

In Figure 8, cells were exposed to 514 nm laser irradiation for only 3 min. For **PS-1**-NPs, such a light dose is enough to kill cells due to the enhanced $^1O_2$ generation efficiency of **PS-1**. While for the control **PS-2**-NPs, we found that approximately 25 min 514 nm (intensity: 12.7 W/cm^2, Light dose: 19.1 KJ/cm^2) laser exposure is required to kill cells incubated with the control **PS-2**-NPs (Figure S3). Meanwhile, the 514 nm laser itself does not cause any obvious cell death (Figure S3 D-F). These results indicated that although the BODIPY dye **PS-2** can also be used as a PDT agent, it needs a much larger light dose to kill cells as compared with the new **PS-1** dye presented in this work. This is why the control NPs did not show any photoxicity under 3 min laser exposure as shown in Figure 8.

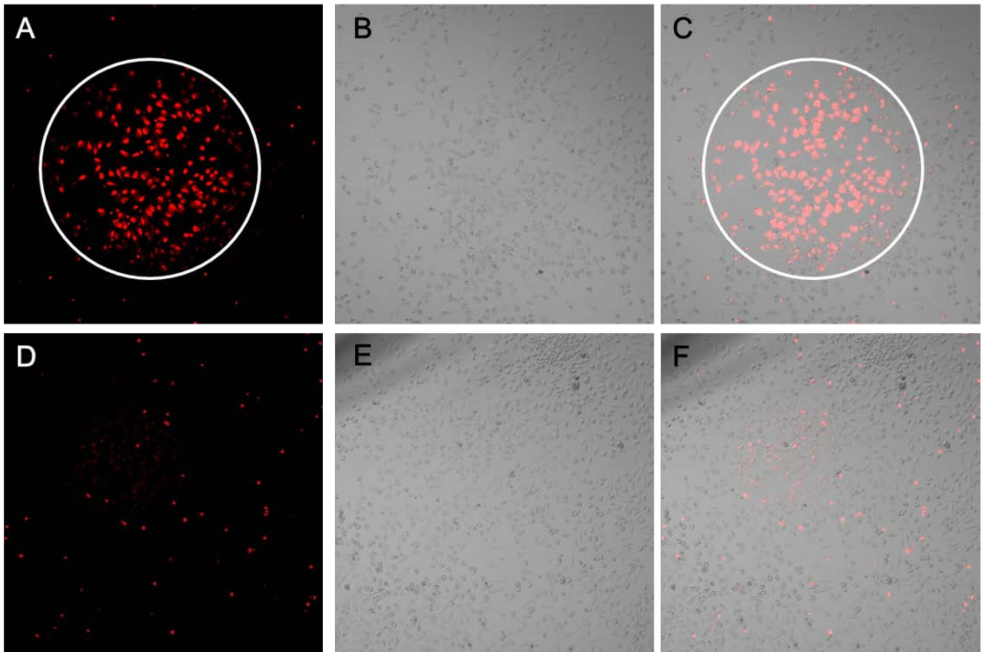

Figure S3. (A-C) HeLa cells incubated with **PS-2**-NPs for 5 h, followed by an exposure to 514 nm laser (intensity: 12.7 W/cm^2) for 25 min. The white circle indicates the light irradiated area. (D-E) HeLa cells incubated with no NPs and exposed to 514 nm laser (12.7 W/cm^2) for 25 min. The left panel is fluorescence channel of Propidium Iodide (PI), the middle panel is bright field images of the HeLa cells, and the right panel is the merged images. Dead cells were stained with PI.
